# Programmed DNA Damage and Physiological DSBs: Mapping, Biological Significance and Perturbations in Disease States

**DOI:** 10.3390/cells9081870

**Published:** 2020-08-10

**Authors:** Sara Oster, Rami I. Aqeilan

**Affiliations:** 1 The Concern Foundation Laboratories, The Lautenberg Center for Immunology and Cancer Research, Department of Immunology and Cancer Research-IMRIC, Hebrew University-Hadassah Medical School, Jerusalem 9112001, Israel; sara.oster@mail.huji.ac.il; 2 Department of Cancer Biology and Genetics, Wexner Medical Center, The Ohio State University, Columbus, OH 43210, USA

**Keywords:** physiological DSBs, DNA repair, meiosis, transcription, BCR, chromosomal translocations, NGS

## Abstract

DNA double strand breaks (DSBs) are known to be the most toxic and threatening of the various types of breaks that may occur to the DNA. However, growing evidence continuously sheds light on the regulatory roles of programmed DSBs. Emerging studies demonstrate the roles of DSBs in processes such as T and B cell development, meiosis, transcription and replication. A significant recent progress in the last few years has contributed to our advanced knowledge regarding the functions of DSBs is the development of many next generation sequencing (NGS) methods, which have considerably advanced our capabilities. Other studies have focused on the implications of programmed DSBs on chromosomal aberrations and tumorigenesis. This review aims to summarize what is known about DNA damage in its physiological context. In addition, we will examine the advancements of the past several years, which have made an impact on the study of genome landscape and its organization.

## 1. Introduction

DNA double-strand breaks (DSBs) are well known for their deleterious effects. Improper repair of these breaks can result in mutations, translocations and even loss of genetic material, which can later lead to tumor formation and cancer progression. There are many exogenous agents that can cause DSBs. For example, ionizing radiation (IR) can target the DNA in two ways, either by directly striking the DNA molecule, mainly causing DSBs, or by water radiolysis, which can result in the formation of reactive oxidative species (ROS) [1]. These radicals can then attack macromolecules in the cell, such as DNA, forming single strand breaks (SSBs) and DSBs in the process. Many other chemicals and genotoxic agents can lead to DNA DSBs, as well [2,3]. Although exogenous agents are a cause of DSBs, the more prevalent source of breaks is endogenous. For example, ROS can be also generated endogenously by oxidative stress brought on by different chemicals, such as H_2_O_2_ or by natural oxygen metabolism [4]. DSBs can also emerge due to replication stress activated by inhibition of DNA synthesis and/or activation of oncogenes [5,6]. Replication stress, resulting in stalling of replication fork, has been proposed to preferentially take place in DNA regions that are under-replicated such as those known as common fragile sites (CFSs). If DSBs at CFSs are misrepaired then genome rearrangements, including copy number variants (CNVs), and genomic instability can occur [7]. It is interesting to note that some of the most common CFSs, such as FRA3B and FRA16D, are encompassed by *FHIT* and *WWOX* tumor suppressor genes, respectively, suggesting that breaks at these genes could infer positive selection and growth advantage [8,9,10,11].

Regardless of its source, breaks that occur in the DNA must be repaired as quickly and as accurately as possible. The most accurate mechanism for DSB repair is homologous recombination (HR). This mechanism occurs during the late S/G2-phase of the cell-cycle and relies on the sister chromatid as a template to repair the broken site. The existing template ensures that no errors will appear at the site of the break, since it will be repaired exactly as the intact template is. The default mechanism for DBS repair is classical nonhomologous end joining (cNHEJ). This mechanism can be activated throughout the entire cell cycle [12,13,14,15]. As the name suggests, in this mechanism the two ends of the broken DNA are joined back together and ligated in place. During this process, Ku70/Ku80 dimer binds to the DNA ends, assisting their protection against end resection, followed by the recruitment of DNA-dependent protein kinase catalytic subunit (DNA-PKcs). The Ku70/Ku80 dimer seems to play an active role in DNA repair choice, by inhibiting HR [16,17]. In most cases, cNHEJ leads to minor and insignificant errors [18,19]. Despite that, if the break resulted in the loss of a piece of DNA or was in the vicinity of another DSB, the simple act of joining and ligation can be between the wrong ends of broken DNA, leading to aberrations of the original DNA sequence (losses and translocations, as previously mentioned). It is essential for all the proteins that participate in the DNA repair to cooperate with each other and perform their role properly. Activation of the NHEJ mechanism inhibits end resection and prevents HR proteins from repairing a given DSBs hence ensuring one mechanism time to operate. When alteration of these mechanisms happens, there are others that can take their place, such as alternative end joining (alt-EJ) or single-strand annealing (SSA) [20]. However, these mechanisms are much less accurate and therefore, more error-prone. The choice of which mechanism would repair a DSB depends on several factors including the cell cycle phase and abundance of DNA repair proteins [20]. Impaired DNA repair will result in mutations and those that are nonlethal of nature and of growth advantage will enable the cells to proliferate and survive contributing to cancer evolution.

In recent years, several lines of evidence support the occurrence of programmed DSBs that constantly occur in the cell with various physiological roles. These DSBs can also be misrepaired, resulting in catastrophic events such as cancer transformation. In this review article, we will discuss recent advances in what is known regarding programmed DNA damage and physiological DSBs and their biological significance.

## 2. Programmed DSBs and Mechanisms of Repair

### 2.1. Meiosis

**Figure 1:**
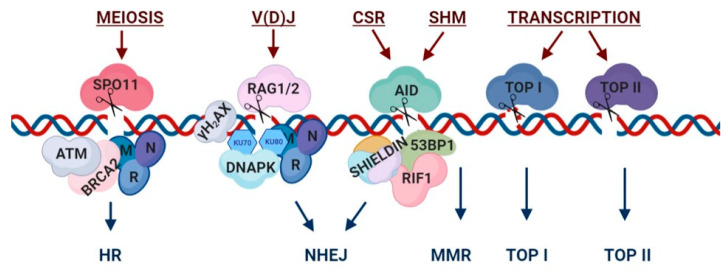
Programmed DNA breaks: nucleases, participating factors and repair pathways. Representative mechanistic view of programmed DNA breaks. During meiosis, SPO11 induces double-strand breaks (DSBs), leading to the recruitment of homologous recombination (HR) repair factors such as ATM, BRCA2 and the MRN complex. DSBs for V(D)J recombination are induced by RAG and are repaired via NHEJ, following γH2AX signaling and the recruitment of the MRN complex and DNA-PKc complex factors. The process of B-Cell Receptor (BCR) diversification including class switch recombination (CSR) and somatic hypermutation (SHM) are initiated by Activation-Induced Cytidine Deaminase (AID). CSR breaks lead to the recruitment of 53BP1, RIF1 and the shieldin complex, which drive repair via NHEJ. SHM breaks are repaired through mismatch-repair (MMR). Transcriptionally induced breaks can be either single- or double-strand breaks and are activated via topoisomerase I (TOP I) or topoisomerase II (TOP II), respectively. This illustration is a simplified version of highly complex break and repair mechanisms. Figure was generated using BioRender tool.

A process that requires DSBs for its execution is meiosis, in which gametes undergo two cell divisions, forming haploid cells [21] (Figure 1). During the prophase I stage of meiosis, homologous chromosomes undergo recombination, allowing genes to ‘cross-over’ and exchange in order to achieve accurate segregation of homologs. Gene diversification in the next generation is another important result of meiosis, as exemplified even in plants [21]. The sites of recombination depend on sequence ‘hot spot’ motifs and require the recognition of zinc-finger protein PRDM9, as shown in mammals [22]. These programmed breaks are mediated via SPO11, a meiosis-specific topoisomerase-like protein whose role is to attack the DNA backbone and break it [23] (Figure 1). After breakage, SPO11 remains bound to the DNA and is recognized by the MRN (MRE11, RAD50 and NBS1), which will subsequently act to remove SPO11, process the DNA ends and later signal for repair [24], suggesting a coupling between processes. In fact, a recent study demonstrates that the MRN complex, or more specifically, NBS1, is crucial for the repair of SPO11-dependant DSBs [25]. This phenomenon is also ATM-dependent [26]. Another role discovered for SPO11 in this context is homologous chromosome pairing, i.e., facilitating the search and coupling of the correct homologous chromosomes during preleptotene, the earliest stage of meiosis [27]. SUN1, a protein whose function is to tether telomeres to the nuclear envelope, is reported in mice to be indispensable to the pairing process as well [27]. Remarkably, in addition to the programmed breakage, meiotic cells carry out mechanisms and checkpoints that ensure a safe number of DSBs. The amount should be enough to allow the recombination to occur. However, too many breaks can be deleterious. The existence of checkpoints can result in meiotic arrest, cell-cycle delay and cell death [28]. In *Saccharomyces cerevisiae*, these mechanisms are controlled by the ATM homolog TEL1 and MRE11 [29]. Repair of meiotic DSBs is achieved by the HR mechanism [30]. In this regard, the template for DNA repair is mainly the homologous chromosome and, less often, the sister chromatid [31]. Two known proteins that initiate the process of meiotic HR are the paralogues RAD51 and DMC1. Currently, the suggested mechanism for meiotic DSBs as proposed by Zhang et al. reveals localization of BRCA2 to the break sites, as shown in mice. This localization is facilitated through interaction with the germline-specific Meiotic Localizer of BRCA2 (MEILB2). Consistent with that, lack of BRCA2 resulted in impaired recruitment of complexes required for the recombination [31]. Surprisingly, this work also pointed out that *Meilb2* mRNA is unusually expressed in some breast cancer cases, and has been recently shown to be directly implicated in cancer [32].

### 2.2. V(D)J Recombination

During lymphocyte development, T and B lymphocytes undergo a process called V(D)J recombination. The purpose of this process is to create diversity in the antigen receptor (TCR or BCR) genes, by creating breaks at specific sequences, which contain V, D or J coding segments [33,34,35]. By utilizing DSBs and repair at the signal sites of the receptor genes, V(D)J recombination diversifies the repertoire of T cell receptors (TCR) in T lymphocytes as well as immunoglobulins (Ig) in B lymphocytes and thus allows for an enhanced ability to recognize a large range of pathogens and antigens [36]. The DSBs are induced via recombination activating genes (RAG) 1 and 2 [37,38]. However, the repair mechanism seems to be more complex (Figure 1). Previous research in the field shows an activation of a DNA damage checkpoint, mediated via p53, in order to regulate the repair at the V(D)J recombination sites and take action if the repair was completed improperly [37]. This is an important step in reducing the likelihood of oncogenesis, which can emerge from translocations between proto-oncogenes and receptor intermediates. In addition, earlier work reveals a dependency of the process on the DNA-dependent protein kinase (DNA-PK), a key factor of the NHEJ repair mechanism [36]. The repair activity of DSBs regulated by this protein is essential for V(D)J recombination. In fact, evidence shows that mutations in the *PRKDC* gene (which encodes for DNA-PKcs) and impaired activity during lymphocyte development results in severe immunodeficiency, through impairment of V(D)J recombination [39]. Moreover, another study demonstrates that in developing T cells, foci of NBS1 (of the MRN complex) and γ-H2AX have been observed in colocalization with the TCR break sites following RAG-dependent cleavage [38]. This, too, insinuates a tight regulation of the DNA damage response (DDR) machinery at the sites of programmed DSBs to minimize oncogenic transformation and allow for proper T cell development. It can be hence postulated that misrepair in the VDJ genomic regions could result in rearrangements and translocations associated with TCR and lymphomagenesis/leukemogenesis.

### 2.3. Class-Switch Recombination (CSR)

Class-switch recombination (CSR) is a process that occurs in mature stimulated B-cells. During CSR, the constant immunoglobulin (Ig) heavy chain genes are broken and then recombined to allow deletion and exchange of the effector Ig gene [40]. At the end of the process, only one of the Ig heavy-chain genes will be expressed in the antibody, which will define the function and capabilities of the antibodies the B-cell produces. The designated antibody function will be one appropriate for the encountered antigen [41]. CSR is initiated by DSBs, introduced via activation-induced cytidine deaminase (AID) [42] (Figure 1). AID deaminates single-strand DNA, causing them to nick and form SSBs, which subsequently turn into DSBs [43]. Following the breakage, the Cμ region is removed and substituted with the next downstream region, utilizing the cell’s DSB repair mechanisms. Previous research in the field revealed that DDR signaling is required for proper CSR. Further investigation focuses on unraveling the complex mechanism, which allows for the successful programmed breakage and repair in CSR. Interestingly, expression of AID is essential for the recruitment of Nsb1/γ-H2AX foci at the sites of breaks related to CSR, suggesting a potential coupling mechanism [44]. CSR is preferentially repaired via NHEJ, as evident by the repair factors, which appear as the CSR break sites. The consensus among many groups reveals 53BP1, the main NHEJ-driving protein, as indispensable to the process [40,41,45,46]. Recently, a protein complex named ‘shieldin’ has been discovered. This complex operates downstream of 53BP1 and is responsible for protecting the ends of broken DNA from resection, allowing for more efficient NHEJ [47]. Loss of components of the shieldin complex results in deficient CSR as well [42]. Another 53BP1-dependant protein whose absence impairs CSR is Rif1. Rif1 acts as part of the mechanism to protect DNA ends from resection following DSBs and thereby assists 53BP1 in driving repair via NHEJ [46]. Consistent with these data, a lack of repair factors, which encourage NHEJ repair leads to DNA repair through other mechanisms, such as alt-EJ, causing a higher rate of chromosomal breaks and resulting in failure to undergo SCR and lack of Ig diversification.

In addition, mature B cells undergo somatic hypermutation (SHM). This process allows for variability of the Ig at the antigen binding area named ‘affinity maturation’ [48], creating a large variety of antibodies. As with CSR, SHM requires AID activity in order to create the DNA lesions that facilitate the rearrangements and mutations and both processes are impaired without it. Nevertheless, it appears that the repair mechanism of SHM differs from what we observed regarding CSR. For example, 53BP1 is not required in the case of SHM [45]. Furthermore, the preferred mechanism for SHM-related breaks is mismatch-repair (MMR), this has been established based on the observation of two MMR genes, *PMS2* and *MSH2*, whose absence impair SHM [49]. MMR occurs after replication and is responsible for fixing wrongly paired nucleotides. This pathway of repair is error-prone, which is an advantage in this case as it allows the variable Ig to mutate constantly and increase the diversity of antibodies. Interestingly, SHM requires replication in order to initiate the repair [50]. On the flip side of genetic heterogeneity, the existence of this purposefully mutagenic process is detrimental and can give rise to tumors [51]. AID overexpression is indeed observed in a number of B-Cell lymphomas [52]. Furthermore, AID-induced mutagenic mismatches has been shown to be indispensable for cause of mutations in B-cell malignancies [53]. Transgenic mouse models overexpressing AID in B-cells have been shown to cooperate with loss of p53 to enhance B-cell lymphomas [54]. Pioneer work by the lab of Michel Nussenzweig has revealed Ig and non-Ig genes to be involved in translocation and rearrangements mediated by the *AID* gene [55]. Altogether, these results imply that perturbations in the programmed DNA damage in B cell development and maturation could result in B-cell malignancies.

### 2.4. Replication and Transcription

Processes that require the opening and separation of the double-stranded DNA, such as replication and transcription, face a significant amount of torsional tension due to the supercoiled state of the DNA [56]. In order to overcome this obstacle, cells express several topoisomerase genes with the purpose of breaking the DNA and subsequently looping it around itself to release the tension [57]. Topoisomerase function is required for transcription initiation, further exemplifying the programmed nature of these breaks [58,59]. The topoisomerases themselves possess the catalytic ability to break the DNA, however, the mechanism of action differs between topoisomerase 1 (TOP1) and TOP2 [60] (Figure 1). TOP1 is active throughout the cell cycle and leads to a single-strand cleavage at the site of the supercoiled DNA. At the site of the break, TOP1 forms a ‘cleavage complex’ with the DNA. Next, TOP1 plays a role in the relaxation of the DNA, achieved by rotating the broken strand around the intact strand several times [61]. Finally, the strand is religated via TOP1 itself. The nucleotide excision repair (NER) pathway may be induced in some cases. The excision repair cross complementing 1 protein-xeroderma pigmentosum group F (ERCC1-XPF) endonuclease collaborates with replication protein A (RPA) in order to repair the nicks caused by TOP1 [62]. TOP2, on the other hand, functions as a dimer, binding either strand of the DNA and together creating DSBs [63]. Relaxation of the DNA is achieved by pulling the two ends apart and passing the same DNA molecule through the gap between the ends [63]. Since the creation of DSBs has more potential for danger, it seems that the repair of TOP2-mediated breaks is more complex. In humans, these breaks are repaired preferentially using the NHEJ repair mechanism [64]. Remarkably, Bermejo et al. show that during S-phase, TOP2 interacts with the HMG protein Hmo1 near transcribed genes as a means to resolve transcription–replication conflicts and maintain genome integrity during replication [65]. Transcription of heat shock related genes [66], serum-induced immediate early genes [67] and nuclear receptor-activated genes [68] have been shown to require DSB generated by TOP2B. Transcription initiation in response to sex hormones has been shown to require TOP2B mediated DSBs, as well. Several reports have implicated stimulation of androgen- and estrogen- receptor target gene expression with the expression and recruitment of TOB2B [69,70,71,72]. These breaks were demonstrated to be both sufficient and necessary for activation of transcription [73]. The function of TOP1 and TOP2 in this context allows the cell to cope with its heavy transcriptional load as well as with the potential dangers that supercoiled DNA poses towards genome integrity.

**Figure 2:**
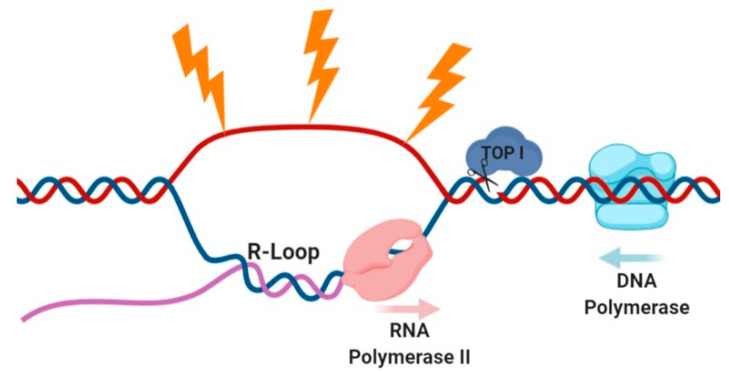
R-Loops are regulated via TOP1 and are subject to breakage through endogenous and exogenous mechanisms. Topoisomerase I (TOP I) relieves transcriptionally induced torsional tension and regulates the formation of DNA breaks (blue)/RNA (purple) hybrids (R-loops). The exposed single-strand DNA segment (red) can be harmed via several processes and develops into a DSB (not shown). Endogenously, breaks can occur due to collisions between transcription machinery (RNA Polymerase II, pink) and replication machinery (DNA polymerase, light blue). The DNA can also be damaged via exogenous stresses, such as IR, UV, oxidative stress and other chemicals. Figure was generated using BioRender tool.

TOP1 has also been shown to regulate the formation of R-loops, a DNA-RNA hybrid formed during transcription [74] (Figure 2). Unscheduled R-loops, formed due to dysregulation of TOP1, are a known cause of genomic instability and chromosomal aberrations, mainly due to the exposure of the non-hybrid single-stranded DNA [75,76]. TOP1 was shown to be involved in activation of specific super-enhancers and recruitment of DSB repair factors, suggesting that TOP1-induced single-strand breaks (SSBs) may develop into DSBs [77,78].

Given that cancer cells can utilize topoisomerase function to allow heavy transcription and replication, recent studies focus on the targeted inhibition of topoisomerases in order to prevent this scenario [60,61,79]. In fact, several topoisomerases poisons such as camptothecin (TOP1 inhibitor) and etoposide (TOP2 inhibitor) are widely used in cancer therapy to target highly proliferation cells with high topoisomerases activity.

## 3. Mapping of DSBs by Next Generation Sequencing

The growing functional significance in physiological DSBs and programmed DNA damage has been further developed and improved due to the development of new methods and technologies, which utilize sequencing capabilities allowing, for the first time, a glimpse at the break pattern of cells, as it appears across the genome (Table 1). Due to advancement in next-generation sequencing (NGS) this has recently become possible. NGS has revolutionized the landscape of genetic research by allowing for millions of strands to be simultaneously sequenced by the means of cell-free library preparation, making the process more effective and comprehensive [80,81]. The range of applications for NGS in research and in diagnostics is boundless, beginning with the ability to recognize mutations in disease and extending toward identifying sites of DNA–protein interactions as well as DNA break spots [82]. For example, in the clinical aspect, NGS methods have made it possible to properly detect and diagnose pathogens [83]. In cancer, NGS can be applied to identify the terrain of a patient’s cancer genome, including mutations, copy-number variations and rearrangements [84]. Such an application can be observed in the work of Dziubańska-Kusibab et al., which identified a mutational signature in colorectal cancer (CRC) that can be traced back to a DNA damaging genotoxin called colibactin, secreted by several *Escherichia coli* strains [85]. This study opens the door to more research regarding the landscape of distinct tumors and the impact of mutational signatures on tumor progression and clinical outcome.

**Figure 3:**
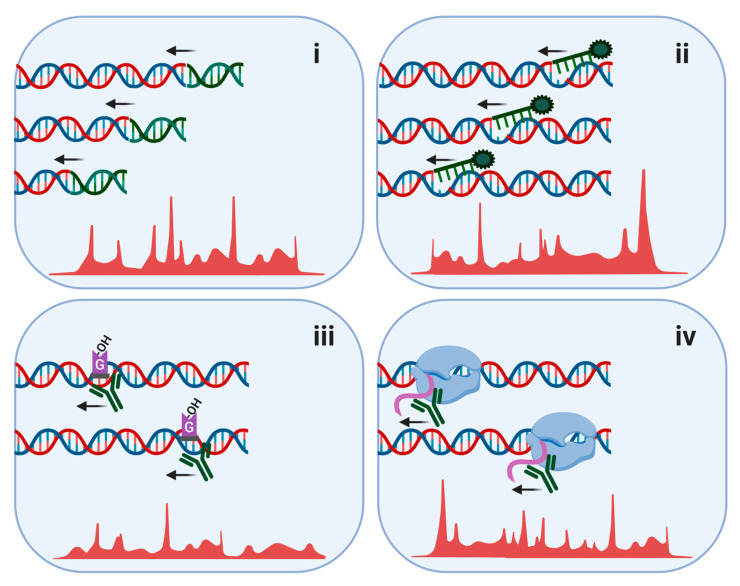
Various NGS methods to recognize different types of DNA damage. (**i**) DSBs (BLESS/BLISS/END-seq/HTGTS), (**ii**) single-strand breaks (SSBs; GLOE-seq), (**iii**) 8-oxodG causing oxidative DNA damage, done by immunoprecipitating the 8-oxodG sites (OxiDIP-seq) and (**iv**) damage at transcriptionally active sites, done by immunoprecipitating RNA strands with radioactive analogues (GRO-seq). Green DNA strands indicate sequencing adapters, used to recognize the damage sites. Arrows indicate sites of sequencing. Red peaks indicate representative sequencing output on the genome. Figure was generated using BioRender tool.

Chromatin immunoprecipitation (ChIP) sequencing (ChIP-seq) was one of the earlier methods to incorporate NGS and has been applied in order to identify sites of protein–DNA interactions [86]. Work done by Hinch et al. demonstrated the use of ChIP-seq in order to understand the roles of RPA, RAD51 and DMC1 in the strand exchange of mammalian meiosis [87]. ChIP-seq can also be utilized to study the chromatin state of genes, by immunoprecipitating and analyzing epigenetic modifications, as demonstrated by Grosselin et al. [88]. Moreover, methods such as BLESS/BLISS or END-seq (reviewed in [89]), which are based on the ligation of sequencing adaptors to the broken DNA ends, have improved our insight into the complex mechanisms of programmed and artificial DSBs. This has allowed for the mapping of breaks that emerge within the context of certain physiological conditions along with detection of factors that have a significant role in these contexts (Figure 3).

BLESS (direct in situ breaks labeling, enrichment on streptavidin and next-generation sequencing), developed by Nicola Crosetto, was the first NGS-based method to directly map the sites of DSBs at the resolution of a single nucleotide. Using their method, Crosetto et al. explored replication stress-induced DSBs [90]. In BLESS, the DSB labeled with a linker that contains a known barcode sequence via ligation. The linker is bound to biotin, which can be later used to pull-down the labeled DSBs using streptavidin beads. The high affinity of biotin to streptavidin allows for specificity of the mapping of the DSBs. The main limitations of this method is that it requires a large number of cells to start with and requires many steps in comparison to newer methods.

**Figure 4:**
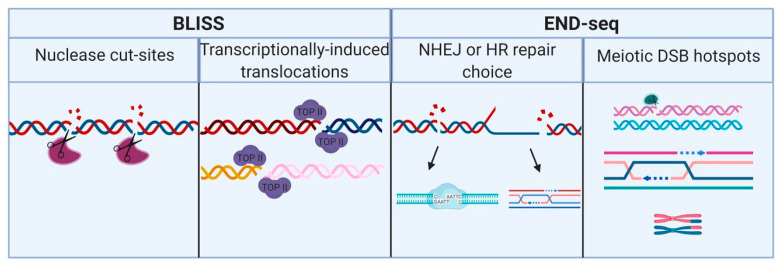
NGS platforms offer new insights and strategies to learn about DNA damage and repair. Methods that can map DNA DSBs, such as BLISS and END-seq, can be utilized for various purposes and multiple studies. Order of panels from the left: First panel, BLISS can be used to recognize cut sites induced by nucleases, for example, CRISPR nucleases Cas9 and Cpf1. Second panel, BLISS has been utilized to identify tumorigenic translocations, such as translocations induced at break-cluster regions induced via Topoisomerase II, which can lead to genomic instability and incorrect religation. Different colors of DNA strands indicate a translocation between strands from remote locations or different chromosomes. Third panel, END-seq can recognize the resection capabilities of DSBs. Therefore, it can be applied to differentiating between breaks that undergo end resection and are thus repaired via HR (right break) as opposed to breaks that are not resected and subsequently repaired via NHEJ (left break). Fourth panel, Due to the ability of END-seq to distinguish break sites that undergo end resection, END-seq can be applied to uncovering meiotic DSB hotspots, which are repaired by HR, and lead to genetic crossovers. Different colors of DNA strands indicate homologous chromosomes of maternal and paternal sources, respectively. Figure was generated using BioRender tool.

Later, the more advanced and efficient BLISS (breaks labeling in situ and sequencing) was developed by the Crosetto group (Figure 3 and Figure 4). Similar to its predecessor, DSBs are labeled with barcoded adaptors in BLISS, as well. However, these adaptors no longer contain bound biotin, but rather, a T7 promotor for in vitro transcription and library preparation. This method also requires a relatively small number of cells to start with. This method has high mapping resolution; however, it is extremely sensitive and therefore creates a high background. Using BLISS, Yan et al. demonstrated the differences in endonuclease specificity between two CRISPR enzymes, Cas9 and Cpf1 [91]. Recent work by Gothe et al. demonstrates utilization of BLISS in order to learn about translocations that occur with the mixed lineage leukemia gene (MLL) by identifying the break-cluster region (BCR) hotspots induced by the TOP2 poison, etoposide. Their findings reveal an enrichment of translocations between MLL and highly transcribed genes at chromatin loop anchors, indicating a mechanism for how genomic instability induced via transcription can lead to tumorigenesis [92].

Dellino et al. set out to identify sites of frequent chromosomal translocations via BLISS, as well. This group reveals the association between DSBs and the release of RNA polymerase II (Pol II) from its pause during elongation in physiological conditions. These breaks occur at sites of certain regulatory elements, such as enhancers, promoters and splice sites, all involved in transcription [93]. Utilization of this advanced methodology is vital for extending our insight into the underlying mechanisms leading to transcriptionally induced translocations, which may eventually become tumorigenic.

Additionally, our laboratory has recently applied the BLISS methodology to characterize the ‘breakome’ in several tumorigenic and non-tumorigenic cell lines. This work linked transcription with repair at the sites of oncogenic super-enhancers [77]. The proposed coupling mechanism supports the heavy transcriptional load of oncogenes, which tumors need to survive and grow [94].

Known contributors to tumor progression are structural variants (SVs), such as amplifications, deletions and translocations. Using BLESS, BLISS and the closely related DSBCapture [95], Ballinger et al. were able to establish, for the first time, a tumor-specific model of SV breakpoints. This model also characterized how these patterns might be affected by the DSB susceptibility signature of different cell types in response to different types of stress, such as replication stress of transcriptional state. These data can shed light on the differences in mutational signatures between cell types and their evolution [96].

Another method, which set to uncover the DSB topography in a sensitive and quantitative manner is END-seq (Figure 3 and Figure 4). Canela et al. introduced a method that can reduce the background and is much more sensitive to low frequency breaks as opposed to the previously mentioned BLESS. Additionally, END-seq visualizes the break’s end resection capabilities. The DSBs are A-tailed and later labeled with adaptors containing a 3′ T overhang and bound to biotin, allowing the breaks to be captured via streptavidin beads, as observed in BLESS. This method requires more cells than BLISS and recurrent breaks in order to identify them. Since it was known that the extent of the end resection at DSB sites affects the choice between NHEJ or HR, this work revealed the RAG-associated DSBs and provided further insight regarding V(D)J, which is known to be repaired via NHEJ [97]. END-seq has also made it possible to learn more about DSBs at recombination signal sequences (RSSs), which allow for V(D)J recombination. Work by Shinoda et al. explored a known model called ‘RAG-scanning’ and uncovered insight into the prediction capabilities of Vκ gene rearrangement and Igκ repertoire [98].

Recently, mapping of ZCWPW1 chromatin biding via CUT&RUN revealed an overlay between the binding sites and meiotic DSB hotspots, mapped via END-seq. ZCWPW1 is a factor that participates in repairing PRDM9-induced DSBs during meiosis. This study, made possible with NGS and END-seq, demonstrated the tight regulation that is ensued due to the collaboration between ZCWPW1 and PRDM9 [99].

Other methods that utilize NGS have emerged as well, mainly differing by means of library preparation and the desirable mapping sites (Figure 3). For instance, high-throughput, genome-wide, translocation sequencing (HTGTS) can detect translocation sites [100,101,102], global run-on and sequencing [103] (GRO-seq) recognizes active transcriptional regulatory elements. In addition, Spo11-oligo-seq, CC-seq and Topo-seq all map topoisomerase cleavage sites [81].

Recently published, OxiDIP-seq exploits NGS in order to detect oxidative damage based on the 8-oxodG marker [104,105] (Table 1). OxiDIP-seq can recognize oxidized DNA fragments, using an 8-oxodG-specific antibody. GLOE-seq, in contrast to BLESS/BLISS or END-seq, can capture SSBs by detecting free 3′-OH ends [106]. These ends are ligated with a biotinylated adaptor, which will subsequently be pulled down via streptavidin. In this method, distinction between the forward and reverse DNA strands is required. As shown in yeast and human cells, GLOE-seq is sensitive and manages to preserve the original DNA nicks, avoiding fragmentation, which, if it occurs, can tamper with the accuracy of the results. A high background due to spontaneous SSBs is a strong limitation to this method.

Together, these methodologies have provided a platform to learn and advance our knowledge regarding the specificity of DSBs in their physiological contexts in a quick and efficient manner.

## 4. New Insights on Misrepair of Physiological DSBs in Cancer Cells

Although DSBs and repair exist as part of the cell’s internal programming, aberrations in many of the factors involved can lead to tumor initiation and progression. It is not surprising, if so, to learn that many of these abnormalities arise due to changes in numerous of the factors mentioned earlier. Programmed DNA damage and breaks that are incorrectly repaired or fused to the wrong break site can give rise to translocation, losses and inversions. All of these are potential drivers of many malignancies [108].

For example, genome instability can occur as a result of AID overexpression, leading to a higher frequency of DSBs at AID target sites and resulting in chromosomal translocations with sites of breaks that arise due to replication stress [109]. These translocations are significant initiators of tumorigenesis. Moreover, RAG has been shown to cleave sequences similar to V, D and J sequences, causing the formation of fusions between antigen receptor loci and other unrelated genes, giving rise to several lymphoid malignancies [109,110].

It is apparent that some individuals can acquire a predisposition to tumor formation, originating from meiotic errors. In most cases, a future loss of the heterozygosity event will initiate the progression [111].

Topoisomerase malfunction can introduce chromosomal aberrations as well. Both replication- and transcription machinery have the potential to collide with a newly produced cleavage complex instigated by active topoisomerases, disrupting their function and preventing efficient religation. Alterations that stem from this mechanism are linked with a number of malignancies [112].

EdUseq enabled the study of mitotic DNA synthesis (MiDAS), occurring due to replication stress. Two recent reports showed that all CFSs, known to be late replicating and commonly deleted in cancer, colocalize with the mapped MiDAS [113,114]. These studies emphasize how replication stress that carries into mitosis can pose a severe threat to genome integrity, which can lead to tumor progression, thus, providing an understanding of how CFSs are implicated in cancer. Identification of these sites can serve as a potential biomarker in the clinic [113,114].

## 5. Concluding Remarks

Looking closely at the various pathways, it is evident that DNA damage occurs as part of evolution and the need for heterogeneity and diversity [115,116]. This notion suggests that the DNA repair mechanisms have emerged in parallel in order to accommodate the break mechanisms. This concept is supported by evidence that nurse sharks have an earlier form of CSR, and yet, all of the shieldin complex proteins, which are integral to the process, are present, suggesting a coevolutionary development [42]. Although DNA repair has evolved in response to programmed DNA damage, it is necessary for the restoration of breaks that are triggered via unprompted processes as well. As previously mentioned, unregulated breaks can emerge due to endogenous processes, such as collisions between transcription and replication or alterations in usually regulated pathways, or exogenous agents, like IR and various chemicals. These breaks are key drivers of many malignancies.

Recent evidence confirms that these pathways do not only drive tumorigenesis. We have previously reported that cancer cells can “hijack” repair pathways in order to maintain the genome’s integrity, which becomes compromised as a result of the heavy transcriptional load introduced in the tumor [77,94]. This observation underscores the importance of understanding how these mechanisms work. Learning which aspects of DDR can be utilized for the tumor’s benefit will allow for producing better therapeutic strategies. For instance, topoisomerase inhibitors, which are used as chemotherapy, trap cleavage complexes on the DNA in order to prevent their religation [117,118].

Interestingly, a recent report revealed a recurrent break clustering pattern in neural progenitor cells, mainly occurring in genes, at CFS [119]. Some of these genes have been shown to be associated with synaptic plasticity, suggesting a possible programmed breakage mechanism in the brain. This study links the DSBs to genetic diversity in neuronal genes and associates repair with cNHEJ as well as with alt-EJ [119]. Although most processes that utilize DNA breaks in order to execute proper function have been known about for decades, this study and others [120,121] (also reviewed in [122,123]) prove that we have yet to uncover them all.

## Figures and Tables

**Table 1:** Methods for identification of DNA breaks via next-generation sequencing (NGS).

Method	Recognizes	Overview	No. of Cells Required	Usages	Limitations	Citations
ChIP-seq	Protein–DNA interactions	Cells are crosslinked and sonicated. Target protein is immunoprecipitated using antibodies linked to beads. Then, DNA is purified and sequenced.	at least 10^6^–10^7^	*The roles of RPA, RAD51 and DMC1 in the strand exchange of mammalian meiosis. *Chromatin state of genes.	*Quality of the antibody. *Cost. *Number of required cells.	[87,88]
BLESS	Sites of DNA DSBs	Cells are crosslinked and labeled by biotin-linked adaptors in-situ. DNA is extracted, sonicated and immunoprecipitated using streptavidin beads. Samples undergo biotin removal and sequencing.	at least 1.5–2×10^6^	*Replication stress-induced DSBs.	*Time-consuming. *Number of cells required. *High background. *Amplification bias.	[90]
BLISS	Sites of DNA DSBs	Cells are crosslinked and labeled by adaptors containing UMI and T7 promotor in-situ. DNA is extracted, sonicated and purified using in-vitro transcription and library preparation. Then, DNA is sequenced.	1×10^6^	*Differences in endonuclease specificity of Cas9 and Cpf1. *Translocations that occur with the mixed lineage leukemia gene (MLL). *Sites of frequent chromosomal translocations. *Linking transcription with repair at the sites of oncogenic super-enhancers. *Tumor-specific model of structural variants (SV) breakpoints.	*Time-consuming. *High background.	[77,91,92,93]
DSBCapture	Sites of DNA DSBs	Cell are fixed and ligated to a biotinylated T-tailed P5 Illumina adapter in order to preserve cohesive ends. DNA is extracted, sonicated and immunoprecipitated using streptavidin beads. Samples undergo biotin removal and sequencing.	1–2×10^7^	*Link elevated gene expression and regulatory sites to DSB.	*Number of cells required.	[95]
END-seq	Sites of DNA DSBs, special focus on resected ends	The DSBs are A-tailed and later labeled with adaptors containing a 3′ T overhang and bound to biotin, allowing the breaks to be captured via streptavidin beads and sequenced.	10^7^	*RAG-associated DSBs, repaired via NHEJ. *DSBs at recombination signal sequences (RSSs). *Overlay between the ZCWPW1 chromatin biding and meiotic DSB hotspots.	*Requires recurrent breaks in order to identify them. *Number of cells required.	[97,98,99]
HTGTS (high-throughput, genome-wide, translocation sequencing)	Translocation sites	Cells are baited to with biotinylated double-stranded DNA for DSBs to translocate with. DNA is later purified, pulled-down using streptavidin beads and sequenced.	10^7^	*DSBs translocations in B-cells were preferentially targeted to transcribed chromosomal regions. *CRISPR/CAS9 modifications.	*Lower sensitivity *Not quantitative due to the possibility of ligation with sequences other than the bait.	[100,101,102]
GRO-seq	Active transcriptional regulatory elements	Addition of 5-Bromo-UTP (BrUTP) to cells is incorporated into actively transcribed RNA. Radiolabeled RNAs are captured using anti-Br-deoxy-U beads. RNA undergoes reverse transcription and subsequently sequenced.	10^7^	*Differentiate between transcriptionally active and inactive regions.	*Time-consuming. *Number of cells required. *High background.	[103]
OxiDIP-seq	Oxidative damage using the 8-oxodG marker	DNA is extracted, sonicated and immuno-precipitated with polyclonal antibodies against 8-oxodG. DNA is then purified, converted from ssDNA to dsDNA and sequenced.	10 μg of genomic DNA per immuno-precipitation	*Coenrichment of 8-oxodG and γH2AX was found within the gene body of transcribed long genes and DNA replication origins. *The study of oxidatively generated DNA damage at gene promoters.	*Distinction between the forward and reverse DNA strands is required.	[104,105]
GLOE-seq	Sites of SSBs	The 3′-OH SSB ends are denatured and ligated with a biotinylated adaptor. Then, DNA is fragmented and captured on streptavidin beads. DNA is then purified, converted from ssDNA to dsDNA and sequenced.	7×10^5^	*Insight into the use of ligases 1 and 3 in human cells. *Map Okazaki fragments. *Can detect asymmetries in spontaneous nicks in yeast and human chromatin.	*Distinction between the forward and reverse DNA strands is required. *High background due to spontaneous SSBs.	[106]
Break-seq	Sites of DSBs	Cells are embedded in agarose plugs. The DNA breaks are End-repaired and labeled using a dATP-bound biotin. Then, DNA is fragmented, captured on streptavidin beads and subsequently sequenced using Illumina TruSeq adaptors.	10^6^ yeast cells	*detection of DSBs caused by replication-transcription conflicts, during exposure and recovery from HU in yeast.	*This method has not been reproduced by other labs.	[107]
